# Paracrine and endocrine actions of bone—the functions of secretory proteins from osteoblasts, osteocytes, and osteoclasts

**DOI:** 10.1038/s41413-018-0019-6

**Published:** 2018-05-24

**Authors:** Yujiao Han, Xiuling You, Wenhui Xing, Zhong Zhang, Weiguo Zou

**Affiliations:** State Key Laboratory of Cell Biology, CAS Center for Excellence in Molecular Cell Sciences, Shanghai Institute of Biochemistry and Cell Biology, Chinese Academy of Sciences, University of Chinese Academy of Sciences, Shanghai, 200031 China

## Abstract

The skeleton is a dynamic organ that is constantly remodeled. Proteins secreted from bone cells, namely osteoblasts, osteocytes, and osteoclasts exert regulation on osteoblastogenesis, osteclastogenesis, and angiogenesis in a paracrine manner. Osteoblasts secrete a range of different molecules including RANKL/OPG, M-CSF, SEMA3A, WNT5A, and WNT16 that regulate osteoclastogenesis. Osteoblasts also produce VEGFA that stimulates osteoblastogenesis and angiogenesis. Osteocytes produce sclerostin (SOST) that inhibits osteoblast differentiation and promotes osteoclast differentiation. Osteoclasts secrete factors including BMP6, CTHRC1, EFNB2, S1P, WNT10B, SEMA4D, and CT-1 that act on osteoblasts and osteocytes, and thereby influenceaA osteogenesis. Osteoclast precursors produce the angiogenic factor PDGF-BB to promote the formation of Type H vessels, which then stimulate osteoblastogenesis. Besides, the evidences over the past decades show that at least three hormones or “osteokines” from bone cells have endocrine functions. FGF23 is produced by osteoblasts and osteocytes and can regulate phosphate metabolism. Osteocalcin (OCN) secreted by osteoblasts regulates systemic glucose and energy metabolism, reproduction, and cognition. Lipocalin-2 (LCN2) is secreted by osteoblasts and can influence energy metabolism by suppressing appetite in the brain. We review the recent progresses in the paracrine and endocrine functions of the secretory proteins of osteoblasts, osteocytes, and osteoclasts, revealing connections of the skeleton with other tissues and providing added insights into the pathogenesis of degenerative diseases affecting multiple organs and the drug discovery process.

## Bone Metabolism: Functions of secretory proteins from bone cells

Proteins secreted from bone cells (osteoblasts, osteocytes, and osteoclasts) regulate the formation of osteoblasts, osteoclasts, and new blood vessels in a paracrine (local hormonal action) manner. The human skeleton is constantly being modeled, and that remodeling involves the removal of old or damaged bone by osteoclasts and its subsequent replacement with new bone by osteoblasts. Osteocytes inhibit osteoblast differentiation and promote osteoclast differentiation. A team headed by Weiguo Zou of the Shanghai Institute of Biochemistry and Cell Biology, University of Chinese Academy of Sciences, reviewed recent progress that has been made regarding the hormonal functions of secretory proteins from osteoblasts, osteocytes, and osteoclasts. The authors highlight the growing awareness of how bone functions as both a paracrine and endocrine (distant hormonal action) organ, which is of great importance in developing treatments for metabolic disorders and degenerative diseases.

## Introduction

The skeleton, accounting for approximately 15% of total human body weight, is one of our largest organ systems in the human body. It has traditionally been considered as a structural organ that provides mechanical support for stature and locomotion in addition to providing protection for vital organs. Bone is also an important reservoir for a number of minerals including calcium, phosphate, magnesium and organic molecules including collagen fibers and amorphous matrix.^1,2^

To facilitate these classical functions and to maintain the integrity of the skeleton, constant remodeling of its architecture and composition occurs throughout an individual’s lifetime. Bone remodeling involves two distinct processes, removal of old or damaged bone by osteoclasts and its subsequent replacement with new bone by osteoblasts.^3,4^ Osteoblasts differentiate from mesenchymal stem cells (MSCs) and comprise 5% of all bone cells, which are responsible for the synthesis of type I collagen and the deposition of mineralized matrix to facilitate the formation of bone.^5,6^ Furthermore, osteoblasts give rise to terminally differentiated osteocytes, the most abundant skeletal cell, which comprise 90% of total bone cells and are embedded in the bone matrix.^4^ These immobilized osteocytes regulate bone composition through translation of mechanical strain into biochemical signals.^7^

Osteoclasts originate from hematopoietic stem cells (HSCs)^8^ and can express vacuolar-ATPases to the ruffled border membrane on the bone surface, where they pump protons into resorption lacunae to dissolve hydroxyapatite. The low pH in the resorption lacunae achieved by the large number of proton pumps activates matrix metalloproteinases (MMPs) and cysteine proteinases to degrade the collagenous bone matrix.^9^ In addition, the blood vessels in the bone can influence bone formation and provide a niche for HSCs that reside in the bone marrow.^10,11^ The involvement of angiogenesis has been reported in bone fracture healing and associated with bone-related diseases including osteoporosis, rheumatoid arthritis and bone cancer.^12,13^

**Fig. 1:**
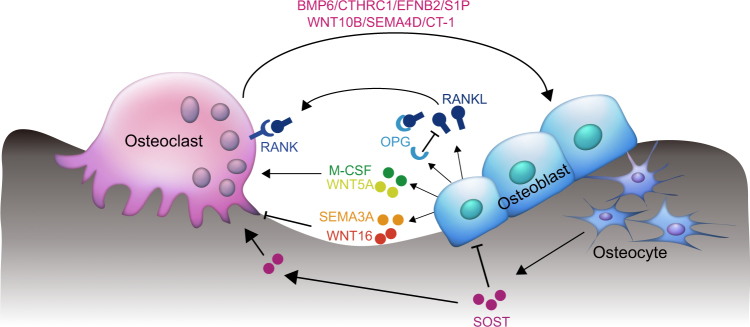
Paracrine actions of osteoblasts, osteocytes, and osteoclasts-derived factors. Molecules secreted by osteoblasts, osteocytes, and osteoclasts influence each other in a paracrine manner to maintain the balance of bone formation and bone resorption. Osteoblasts activate osteoclast formation by expressing M-CSF, RANKL, and WNT5A and inhibit osteoclast activity through OPG, a decoy receptor of RANKL, SEMA3A, and WNT16. Osteocyte-derived SOST inhibits osteoblast differentiation and stimulates osteoclastogenesis. Osteoclasts also secrete coupling factors such as BMP6, CTHRC1, EFNB2, S1P, WNT10B, SEMA4D, and CT-1 to act on osteoblasts and osteocytes and thereby influence bone formation.

Within the microenvironment niche, osteoblasts, osteocytes, and osteoclasts synthesize and secrete paracrine signaling molecules, including growth factors, cytokines and chemokines to maintain the remodeling and architecture of skeleton. Molecules secreted by osteoblasts and osteocytes which affect osteoclastogenesis include monocyte/macrophage colony-stimulating factor (M-CSF),^14–16^ receptor activation of NF-кB ligand (RANKL),^17–19^ anti-osteoclastogenic factors such as osteoprotegerin (OPG), a decoy receptor of RANKL^20–22^ and Semaphorin 3A (SEMA3A),^23^ Wnt gene family 5A (WNT5A) and 16 (WNT16) (Fig. 1). Osteocytes have been reported to secret sclerostin (SOST) which inhibits osteoblast differentiation and subsequently bone formation in a paracrine manner^24^ (Fig. 1). Osteoclast-derived factors, including bone morphogenetic protein 6 (BMP6),^25^ collagen triple helix repeat containing 1 (CTHRC1),^26^ EphrinB2 (EFNB2),^27^ Sphingosine 1-phosphate (S1P),^28^ Wnt gene family 10b (WNT10B),^29^ Semaphorin 4D (SEMA4D), and Cardiotrophin-1(CT-1), affect the differentiation and/or functions of osteoblasts and osteocytes (Fig. 1).

**Fig. 2:**
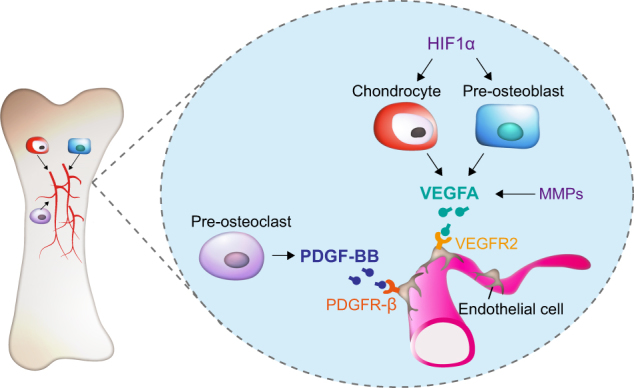
Regulation of angiogenesis by secretory factors from different bone cells. Bone formation and bone resorption are coupled by the process of angiogenesis. Pre-osteoblasts and chondrocytes-derived vascular endothelial growth factor A (VEGFA) can promote proliferation, survival and migration of endothelial cells (ECs), which express VEGF receptor 2 (VEGFR2). Hypoxia-inducible factor 1-α (HIF1α) can induce VEGFA expression in chondrocytes and pre-osteoblasts. Matrix metalloproteases (MMPs) can affect VEGF release from the ECM. Pre-osteoclast-secreted platelet-derived growth factor-BB (PDGF-BB) can induce Type H vessel formation and thereby stimulate bone formation.

The paracrine actions of secretary factors from osteoblasts, osteocytes and osteoclasts allow balancing of bone formation with bone resorption, processes which are also coupled to angiogenesis. Vascular endothelial growth factor A (VEGFA), derived from pre-osteoblasts and chondrocytes, is a major pro-angiogenic factor that can promote proliferation, survival, and migration of endothelial cells (ECs), a major cell population that expresses VEGF receptor 2 (VEGFR2) (Fig. 2).^30–32^ Platelet-derived growth factor-BB (PDGF-BB), also an angiogenesis factor secreted by pre-osteoclasts, can induce Type H vessel formation and thereby stimulating bone formation (Fig. 2).^33^ Briefly, these paracrine factors produced by bone cells are released into the extracellular environment and act on nearby cells to maintain bone homeostasis.

**Fig. 3:**
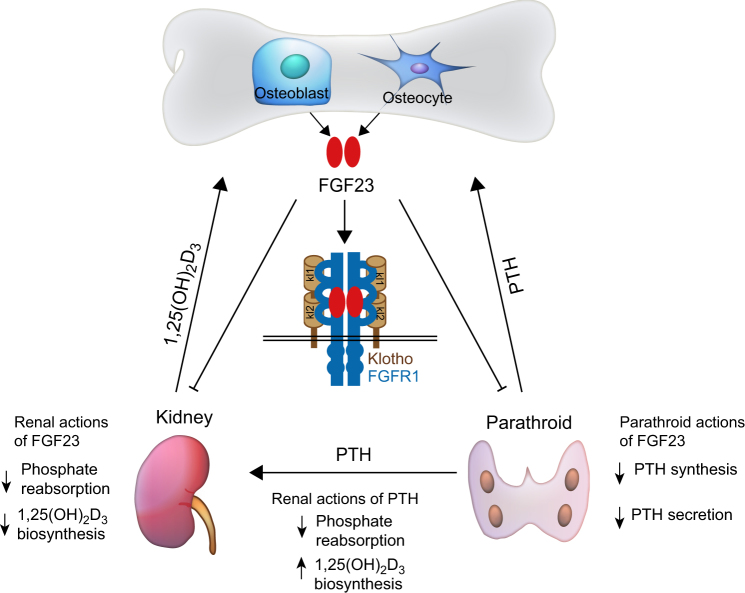
Bone-derived FGF23 regulates phosphate metabolism. FGF23, synthesized by osteoblasts and osteocytes, inhibits phosphate resorption and suppresses the production of 1,25(OH)_2_D_3_ through its binding to a complex of FGFR1 and the co-receptor Klotho in the kidney. FGF23 also suppresses PTH synthesis and secretion in a Klotho-dependent fashion in the parathyroid. Synthesis and secretion of FGF23 by osteoblasts and osteocytes are positively regulated by 1,25(OH)_2_D_3_ and PTH. PTH derived from parathyroid can downregulate phosphate resorption and 1,25(OH)_2_D_3_ production in the kidney.

**Fig. 4:**
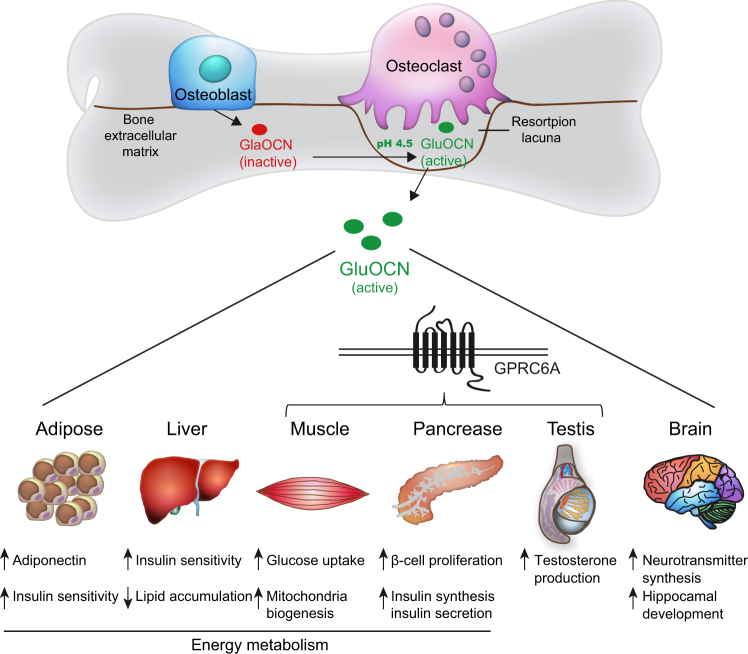
OCN is a bone-derived multifunctional hormone. OCN is C-carboxylated (GlaOCN) and secreted by osteoblasts into bone extracellular matrix (ECM). The acidic pH (~4.5) in the resorption lacunae formed by osteoclasts decarboxylates GlaOCN into undercarboxylated active osteocalcin (GluOCN), which enters the circulation to act as a hormone. GluOCN regulates energy metabolism via enhancement of glucose uptake in muscle, insulin production in the pancreas, insulin sensitivity in the liver and adipose tissue, upregulation of adiponectin expression in adipose tissue and promotion of β-cell proliferation in the pancreas. In addition, OCN promotes male fertility by stimulation of testosterone synthesis in Leydig cells which improves cognitive function of the brain through an increase in neurotransmitter synthesis and facilitation of hippocampus development. Notably, OCN functions in testis, pancreas and muscle through its binding to the receptor GPRC6A while receptor(s) of OCN in the brain, adipose, and liver still require identification.

**Fig. 5:**
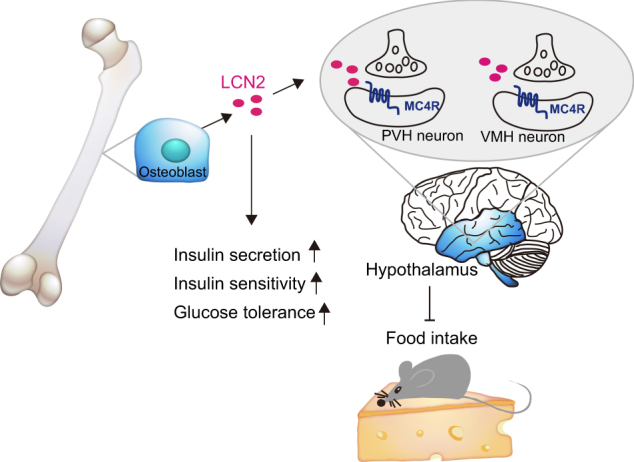
Osteoblast-derived LCN2 suppresses food intake. LCN2 is secreted by osteoblasts and crosses the blood–brain barrier to accumulate in the hypothalamus, where it binds to its receptor MC4R in the hypothalamic neurons of the paraventricular nucleus (PVN) and ventromedial hypothalamus (VMH) and activates MC4R-dependent anorexigenic signaling. In addition, LCN2 also directly regulates glucose tolerance, insulin sensitivity and insulin secretion.

In addition to its structural role, the skeleton has also been recognized as an endocrine organ for the hormonal modulation of energy homeostasis. Bone-derived secretory factors comprise an important endocrine system that is finely orchestrated with other organs to ensure homeostatic balance and health.^3^ Bone-derived fibroblast growth factor 23 (FGF23) was discovered from the information in the genetic analyses of human familial disorders of phosphate homeostasis.^34^ Subsequent studies have recognized that it is principally secreted by osteoblasts and osteocytes in the skeleton and plays important roles in modulating phosphate homeostasis by inhibiting phosphate reabsorption and 1,25-dihydroxyvitamin D_3_[1,25(OH)_2_D_3_] production in the kidney and suppressing parathyroid hormone (PTH) synthesis in the parathyroid gland which reduces the circulating phosphate levels^35,36^ (Fig. 3). Through a series of murine genetic manipulations and clinical observations including disease symptoms and drug side effects, osteocalcin (OCN) has been identified as an additional bone-derived endocrine hormone that regulates the biological processes of multiple organs including bone, adipose, liver, muscle, pancreas, testes, and brain^1,6,37–44^ (Fig. 4). Moreover, the latest research demonstrates that osteoblast-derived lipocalin-2 (LCN2) inhibits food intake by binding to the melanocortin 4 receptor (MC4R) in the hypothalamus and regulates glucose tolerance, insulin sensitivity, and insulin secretion to maintain glucose homeostasis^45^ (Fig. 5).

From an evolutionary perspective, the skeleton produces hormones to regulate skeletal development and remodeling and cooperates with other endocrine organs to control the homeostasis of phosphate and calcium metabolism and thereby maintaining energy balance, which indicates that bone is a strongly selected survival factor beyond its mechanical roles.^3^ The growing awareness that bone is both a paracrine and endocrine organ will broaden our understanding about the pathogenesis of bone involved metabolic disorders and degenerative diseases. The identification of further paracrine and endocrine factors in bone will also shed light on the development of novel pharmaceutical treatments for these diseases.^2–4,46,47^

## Paracrine actions of the osteoblast-derived and osteocyte-derived factors

### M-CSF

M-CSF was originally defined as a hematopoietic cell growth factor which promoted macrophages from bone marrow progenitors to form colonies in semisolid media and was produced constitutively by a variety of cells such as macrophages, ECs, fibroblasts, osteoblasts etc.^48,49^ Then in 1986, M-CSF was first reported to stimulate osteoclast-like cell formation in long-term culture.^15^ Follow-up studies indicated that it was not only indispensable for the proliferation and differentiation of osteoclast progenitors but was also required for the survival, motility and spreading of osteoclasts^14,16^ (Fig. 1). Osteoblasts and bone marrow stromal cells were subsequently shown to be the principal source of M-CSF in the bone microenvironment, producing both the soluble and membrane-bound form of M-CSF.^50^ The role of M-CSF in osteoclastogenesis in vivo has been confirmed using an osteopetrotic (*op/op*) mutant mouse, a thymidine insertion in the *Csf-1* gene resulted in M-CSF deficiency, which led to decreased numbers of macrophages and osteoclasts.^51–53^ Delivery of recombinant M-CSF to *op/op* mice resulted in an increased number of osteoclasts, which led to amelioration of the osteopetrotic defect.^53,54^ Furthermore, osteoblast-specific targeting with soluble M-CSF to the *op/op* mice rescued the osteopetrotic phenotype.^55^ Taken together, these findings demonstrate that osteoblast-derived M-CSF is critical for osteoclast formation.

### RANKL

RANKL, also known as TNFSF11, TRANCE, OPGL, and ODF, is expressed largely in bone, lymphoid tissue, stromal cells, and activated T lymphocytes.^18,19^ RANKL was initially identified as a cytokine produced by T cells which played an essential role in the regulation of the T cell-dependent immune response and appeared to be an important regulator of the interaction between T cells and dendritic cells.^56,57^ An in vitro study demonstrated that RANKL was required to induce osteoclast-like (OCL) cell formation in the presence of permissive levels of M-CSF, suggesting its potential role in regulating osteoclast differentiation.^58^ Further studies showed that RANKL was indispensable for the formation, fusion, activation, and survival of osteoclasts by binding to its receptor, receptor activator of the NF-κB (RANK), on osteoclasts and its precursors.^57–60^ Mice with a disrupted *Opgl* gene, which encodes RANKL, demonstrated severe osteopetrosis, completely lacking osteoclasts because *Opgl*^*−/−*^ osteoblasts and osteocytes do not support osteoclastogenesis.^17^ Conversely, *Opgl* transgenic mice with excessive production of RANKL displayed extreme osteoporotic phenotypes^61^ (Fig. 1). Hence, for osteoporosis and related disorders, RANKL signaling through which bone resorption is regulated^62^ has become a popular target. The RANKL monoclonal antibody, Denosumab, has shown promise in the treatment of osteoporosis.^63^

### OPG

OPG is a member of the TNF receptor superfamily and also known as osteoclastogenesis inhibitory factor (OCIF), TNFRS member 11B (TNFRS11B), and TR1.^20–22,59^ It was identified as a secreted glycoprotein synthesized by several kinds of cells including osteoblasts, B lymphocytes, and articular chondrocytes.^20,22,59^ An in vivo study with *Opg* transgenic mice revealed that overexpression of OPG led to profound yet nonlethal osteopetrosis because of decreased numbers of osteoclasts. An in vitro study revealed that OPG inhibited osteoclast differentiation from their precursors.^20,21^ Further study showed that OPG acted as a soluble decoy receptor of RAKNL, the main function of OPG being to antagonize the effects of RANKL and interrupt the crosstalk between osteoblasts and osteoclasts^64^ (Fig. 1).

### SEMA3A

SEMA3A, also known as C-Collapsin-1, H-SEMA III, M-SEMD, R-SEMA III, and SEMA-Z1A, is the first identified vertebral semaphorin and originally characterized as a diffusible axonal chemorepellent that prevented growth and branching of axons into inappropriate areas.^65^ SEMA3A has been extensively studied in the nervous system and is now also recognized to be involved in bone remodeling.^65,66^ Additional to the known anti-osteoclastogenesis factor OPG, SEMA3A was also identified as an osteoblast-secreted inhibitor observed in conditioned media from OPG-deficient osteoblasts by mass spectrometry^23^ (Fig. 1). In vivo studies demonstrated that *Sema3a*^*−/−*^ mice exhibited a severe osteopenic phenotype both in trabecular and cortical bones due to increased numbers of osteoclasts and decreased numbers of osteoblasts.^23^ Furthermore, SEMA3A regulates osteoclast differentiation via binding to neuropilin-1 (NRP1), demonstrated by mutant Nrp1 mice, lacking the Sema-binding site (Nrp1^Sema−^), which phenocopy *Sema3a*^*−/−*^ mice.^23^

### WNT5A and WNT16

It is known that Wnt signaling plays a crucial role in regulating bone homeostasis. Wnt ligands orchestrate critical events important for the activity of bone cells by engaging various WNT receptor complexes then inducing different signaling cascades.^67,68^ It has been reported that WNT5A secreted by osteoblasts enhance osteoclastogenesis through the receptor tyrosine kinase-like orphan receptor 2 (Ror2) which is expressed by osteoclast precursors^69^ (Fig. 1). Wnt5a–Ror2 signal stimulates RANK expression in osteoclast precursors by promoting the phosphorylation of JNK and recruiting c-Jun to the promoter of *Rank* gene, thereby enhancing RANKL-induced osteoclastogenesis.^69,70^ In contrast, osteoblast-derived WNT16 has been shown to inhibit osteoclast formation by both directly interfering with osteoclast differentiation via RANK signaling and indirectly increasing *Opg* expression in osteoblasts through both canonical and non-canonical Wnt signaling^71^ (Fig. 1). Conditional deletion of WNT16 in osteoblast lineage increases fracture susceptibility^71^ and WNT16 has consistently been demonstrated to be a major determinant of non-vertebral fracture risk in humans.^72,73^ The finding that osteoblast-derived WNT16 is an anti-osteoclastogenic factor provides new avenues for prevention or treatment of fractures.

### SOST

The human genetic bone disorder, sclerosteosis, provides an insight into the role of SOST in bone regulatory processes. Sclerosteosis is a disease characterized by high-bone mass due to the lack of SOST, encoded by the *Sost* gene.^74,75^ SOST is strongly expressed in osteocytes, exhibiting significant inhibition of osteoblast activity and bone formation in vivo^24^ (Fig. 1). Osteocytes sense mechanical stress and specifically express SOST that inhibits osteoblast differentiation through antagonism of the canonical Wnt pathway.^76^ Upon binding of a Wnt ligand to its membrane-bound receptor complex, which comprises Frizzled and low-density lipoprotein receptor-related protein 5/6 (LRP5/6), canonical β-catenin-dependent signaling is activated.^77^ SOST binds to the extracellular domain of LRP5/6 in osteoblasts and disrupts Wnt-induced activation of bone formation related genes.^76^

Another LRP5/6 antagonist, Dickkopf-1 (DKK1), is also expressed by osteocytes, however, is not as highly selective as SOST. Transgenic overexpression of DKK1 induces severe osteopenia^78^ and deletion of a single allele of the Dkk1 gene leads to increased bone formation and bone mass.^79^ In addition to its anti-anabolic role, SOST has also been found to stimulate osteoclastogenesis in a RANKL-dependent manner^80^ (Fig. 1) and induce the release of bone mineral through the mediation of acidification of the extracellular matrix (ECM) by upregulation of carbonic anhydrase 2 (CA2), cathepsin K (CTSK), and tartrate-resistant acid phosphatase (TRAP) expression in osteoclasts^81^ (Fig. 1). SOST shows the key role that osteocytes have in mediating the molecular mechanisms involved in adaptive bone remodeling, balancing the bone resorption–formation axis.^74^ Antibodies against SOST and DKK1 are developed as a promising, novel treatment for osteoporosis.^77^

### VEGFA

VEGFA plays a major role in angiogenesis and is secreted by hypertrophic chondrocytes and pre-osteoblasts in bone^82,83^ (Fig. 2). Conditional deletion of *Vegfa* in chondrocytes impairs both vessel invasion and chondrocyte survival.^84,85^ In osteoblast lineage cells, overexpression of VEGFA can enhance both bone angiogenesis and osteogenesis through activation of Wnt/β-catenin signaling.^86^ It has been reported that VEGFA can bind VEGFR2 in ECs, thereby stimulating EC migration and proliferation^31,32^ (Fig. 2). There are several angiogenesis regulators functioning through VEGFA. For instance, Hypoxia-inducible factor 1-α (HIF1α) can regulate VEGFA expression in hypertrophic chondrocytes and osteoblasts and hence promote angiogenesis in bone^87^ (Fig. 2). MMP-mediated ECM remodeling is essential for angiogenesis and osteogenesis^88,89^ (Fig. 2). It has been reported that MMP9 plays an important role for VEGF release from the ECM.^90^ In particular, MMP9-deficient mice show reduced vascularization during bone development.^91^ While administration of exogenous VEGF can rescue endochondral ossification deficiency in *Mmp9* knockout mice.^92^ Taken together, these findings show that VEGFA derived from hypertrophic chondrocytes and osteoblasts is a master regulator of angiogenesis in bone.

## Paracrine actions of osteoclast-derived factors

### BMP6

It has been reported that systemic administration of BMP6 in ovariectomized rats can increase bone formation and decrease bone resorption.^93^ BMPs play important roles in promoting the recruitment, proliferation and differentiation of osteoblasts at bone resorption sites. It has been reported that BMPs 2, 4, 6, and 7 are expressed in osteoclasts, as determined by immunocytochemistry and in situ hybridization, suggesting a possible direct role for osteoclasts in promoting bone formation via the synthesis and secretion of BMPs^94^ (Fig. 1). BMP6 has since been identified using the affymetrix microarray as an osteoclast-derived coupling factor, which has been proved to recruit osteoprogenitors to the sites of bone remodeling and stimulate bone formation.^25^ In addition, the deletion of the BMP receptor type IA (*Bmpr1a*) gene in an osteoclast-specific manner using *Ctsk-cre* led to increased bone formation, suggesting that the loss of BMP signals within osteoclasts increases osteoblast activities.^95^ However, it has been suggested that *Ctsk-cre* is expressed in chondrocyte progenitors, and possibly osteoblast progenitor cells. This conclusion suggested by *Bmpr1a* conditional knockout mice using *Ctsk-cre* might require further clarification.^96^

### CTHRC1

CTHRC1 was originally isolated from injured arteries.^97^ Expression of CTHRC1 was further found to be clearly elevated in active, but not inactive, osteoclasts. The low-bone-mass-phenotype of *Cthrc1*-null mice and the high-bone-mass-phenotype of *Cthrc1* transgenic mice indicated that CTHRC1 was a positive regulator of osteoblastic bone formation.^98^ Osteoclast-specific deletion of *Cthrc1* resulted in reduced bone formation due to impaired coupling processes (Fig. 1). In brief, CTHRC1 has been demonstrated to be an osteoclast-secreted coupling factor that regulates bone remodeling.^26^

### EFNB2

EFNB2 is encoded by the nuclear factor of activated T cells cytoplasmic 1 (NFATc1) target gene *Efnb2* and expressed in osteoclasts, while its receptor EphB4 is expressed in osteoblasts. A combination of in vitro and in vivo approaches demonstrated that EFNB2–EphB4 bidirectional signaling linked the suppression of osteoclast differentiation to the stimulation of bone formation, which may regulate the transition from a bone resorption pattern to a bone formation pattern^27^ (Fig. 1).

### S1P

S1P is a phosphorylated sphingosine catalyzed by sphingosine kinase 1 (SPHK1), a lipid kinase expressed in osteoclasts. Upregulated SPHK1 expression and increased S1P production and secretion have been observed in a bone marrow-derived macrophage model system following RANKL stimulation. Addition of S1P to the BMM/osteoblast co-culture system greatly increased osteoclastogenesis by increasing RANKL expression from osteoblasts. These results indicated that SPHK1 and S1P play important roles in the regulation of osteoclastogenesis and in the communication between osteoclasts and osteoblasts.^28^ Affymetrix microarrays performed by Pederson *et al* also identified S1P as an osteoclast-derived coupling factor^25^ (Fig. 1). Other genetic evidence has shown that deletion of *Ctsk* in osteoclasts enhances bone formation in vivo by increasing the generation of osteoclast-derived S1P, which can be inhibited by S1P receptor antagonist.^99^

### WNT10B

Along with BMP6 and S1P, WNT10B has been identified as an osteoclast-derived coupling factor through which osteoclasts may recruit osteoblast progenitors to the site of bone remodeling.^25^ The fact that TGF-β1 increases osteoclast production of WNT10B, but not BMP6 or S1P in osteoclasts, to promote osteoblastic cell mineralization suggests that WNT10B contributes to the enhanced coupling of osteoclasts to osteoblasts^29^ (Fig. 1).

### SEMA4D

SEMA4D is an axon-guidance molecule belonging to the Semaphorin family, expressed exclusively by osteoclasts, but not by osteoblasts.^100^ SEMA4D inhibits bone formation (Fig. 1) by modulating osteoblast motility and suppressing insulin-like growth factor-1 (IGF-1) signaling through binding to its receptor Plexin-B1 on osteoblasts and activation of the small GTPase RhoA. Injection of a SEMA4D-specific antibody markedly prevented bone loss in a postmenopausal osteoporosis model by promoting bone formation without affecting bone resorption, suggesting SEMA4D could be a new and potentially effective target for bone-increasing drugs.^100^

### CT-1

CT-1 is a member of the interleukin-6 (IL-6) family and signals through GP130 and the LIF receptor (LIFR).^101^ It is expressed by osteoclasts and is essential for normal bone resorption (Fig. 1). And it has also been proved that osteoclasts secreted CT-1 had a paracrine role, as a “coupling factor”, acting on osteocytes, osteoblasts, and their precursors to stimulate bone formation during remodeling.^102^ The mechanism of CT-1 signaling through GP130 and LIF-R was postulated through the strikingly parallel bone phenotypes observed in *Ct-1*^*−/−*^, *Gp130*^*−/−*^, and *Lif-r*^*−/−*^ mice.^103,104^ Increased expression of C/EBP induced by CT-1 indicates a mechanism for this paracrine effect, whereas C/EBP acts synergistically with Runx2 to activate osteocalcin transcription.^102^

### PDGF-BB

Platelet-derived growth factor-BB (PDGF-BB) induces migration of endothelial progenitor cells (EPCs) and hence angiogenesis.^105^ Furthermore, PDGF-BB secreted from osteoclast stimulates migration and osteogenic differentiation of MSCs.^106^ It was reported that PDGF-BB derived from pre-osteoclasts could induce Type H capillary formation coupling osteogenesis during bone modeling and remodeling^33^ (Fig. 2). There are two subtypes of capillaries in bone according to their marker expression and functional characteristics: type H and type L. Type H capillaries express high levels of endomucin (EMCN) and CD31 and are located in the metaphysis and endosteum surrounded by osteoprogenitor cells. Type L capillaries, on the other hand, are mainly present in the medullary region with lower levels of EMCN and CD31. Type H capillaries can couple angiogenesis and osteogenesis during development.^10,107^

## Endocrine actions of osteoblast-derived and osteocyte-derived factors

### FGF23

The hypothesis that phosphate metabolism was regulated by a secretory factor came from the clinical observation that patients with a phosphate-wasting disease could not be rescued by transplanting a healthy kidney, impling that the cause of phosphate-wasting might originate from another organ.^108^ The *Fgf23* missense mutation was then identified in patients with autosomal dominant hypophosphatemic rickets (ADHR), an inherited disorder involving disturbed in phosphate homeostasis. This fact provides insight into the possibility that FGF23 physiologically regulates phosphate metabolism.^34^ FGF23 is found to be produced by normal and fibrous dysplasia (FD) forms of bone osteoprogenitors and osteocytes in vivo and in vitro^109^ (Fig. 3). In addition, the production of FGF23 by dysplastic bone plays a crucial role in the renal phosphate-wasting syndrome associated with FD.^109^ These studies make FGF23 a unique member of the FGF family as it functions as a hormone that derives from bone and regulates phosphate metabolism in the kidney, which is critically important for bone health.^35,110,111^

FGF23, synthesized by osteoblasts and osteocytes, inhibits phosphate reabsorption in the renal proximal and distal tubules of the kidney and suppresses the production of 1,25-Dihydroxyvitamin D3 [1,25(OH)_2_D_3_] through inhibition of 1a-hydroxylase. FGF23 regulates phosphate reabsorption through binding to a complex of FGFR1 and the co-receptor Klotho,^112^ which is reported to be essential for endogenous FGF23 function (Fig. 3). Klotho can significantly enhance the ability of FGF23 to induce phosphorylation of FGF receptor substrates and activate FGF signaling.^112–114^ In addition, the parathyroid is also a target organ of FGF23 as FGF23 suppresses PTH synthesis and secretion in vitro and in vivo in a Klotho-dependent fashion.^115^ However, FGF23 synergizes with PTH to increase renal phosphate excretion by reducing reabsorption of the sodium-phosphate in the proximal tubules^36^ (Fig. 3).

Human genetic disorders and genetically engineered mice have accelerated the understandings of the regulation of phosphate homeostasis by PTH, 1,25(OH)_2_D_3_ and FGF23. Synthesis and secretion of FGF23 by osteocytes are positively regulated by 1,25(OH)_2_D_3_ and serum phosphorus.^116^ In turn, FGF23 inhibits the synthesis of 1,25(OH)_2_D_3_ and negatively regulates the secretion of PTH from the parathyroid glands. 1,25(OH)_2_D_3_ expression is upregulated by PTH and downregulated by increased serum phosphate and FGF23 levels.^117^ 1,25(OH)_2_D_3_ acts through VDR/RXR dimers to stimulate FGF23 synthesis and secretion by osteocytes.^36^ PTH increases osteoblast activity, inhibits renal phosphate reabsorption and stimulates 1,25(OH)_2_D_3_ synthesis by binding to its receptor PTHR(Fig. 3).

### OCN

OCN, also known as BGLAP, was initially discovered by two independent groups, isolated from calf and chicken’s bone, respectively.^118,119^ It is the most abundant osteoblast-specific non-collagenous protein and is a determinant of bone formation.^37,120^ Evidences accumulated over the past decade show that OCN acts as an endocrine hormone on multiple organs, including adipose, liver, muscle, pancreas, testis, and brain.^1,6,37–44^ An important difference between OCN and FGF23 is that FGF23 regulates phosphate metabolism, a process intimately linked to bone health itself, while OCN has many more functions.^1,114,121^

### Modification and regulation of OCN

OCN is first synthesized as a prohormone (pro-OCN), prior to cleavage by an intracellular proprotein convertase called Furin to be matured in osteoblasts.^122^ Before secretion, OCN is C-carboxylated on its glutamate residues in the endoplasmic reticulum (ER) of osteoblasts, by c-glutamyl carboxylase (GGCX) with vitamin K as a cofactor. These post-translational modifications increase the affinity of OCN for calcium (Ca^2+^) and hydroxyapatite crystals, the principal mineral composition of the bone ECM, and hence facilitate the trapping of the majority of secreted c-carboxylated osteocalcin (GlaOCN) into ECM to form the most abundant non-collagen peptides.^120^ The acidic environment generated by osteoclasts during the bone resorption process promotes decarboxylation of GlaOCN into undercarboxylated osteocalcin (GluOCN), decreasing its affinity for bone matrix and therefore promoting its release into the circulation. It has been shown that an acidic pH (~4.5) is the only mechanism known to achieve decarboxylation of proteins. Although both GlaOCN and GluOCN are detectable in the circulation, it is only the GluOCN, which has been demonstrated to function as a hormone in regulating energy metabolism^1,123^ (Fig. 4). Consequently, mice with increased osteoclast activity display increased circulating levels of bioactive GluOCN, improved glucose tolerance and insulin sensitivity, whereas mice lacking osteoclasts have decreased levels of bioactive GluOCN and glucose tolerance.^124^ In addition, insulin signaling in osteoblasts leads to reduced OPG expression and increased osteoclast activity, resulting in the release of bioactive GluOCN.

### Regulation of energy metabolism by OCN

The hypothesis that bone-derived OCN regulates glucose metabolism originates from the studies of *Ocn*-null mice, which exhibit an accumulation of abnormal quantities of body fat and reduced peripheral insulin sensitivity in addition to impaired glucose metabolism. *Ocn*-null mice also displayed liver steatosis and inflammation in white adipose tissue.^41,121,123^ Consistent with these observations, injections of recombinant OCN in lean or obese mice resulted in increased energy expenditure, reduced fat mass, improved insulin sensitivity and prevented liver steatosis.^41,42^ *Esp*, also known as *Ptprv*, is a gene encoding osteotesticular protein tyrosine phosphatase (OST-PTP). It was shown to negatively regulate insulin receptor signaling, decrease bone resorption, suppress the decarboxylation of GlaOCN and therefore reduce the quantity of active GluOCN.^42,125^ It was demonstrated that OST-PTP plays an important role in regulating glucose metabolism because mice lacking *Esp* (*Esp*^*−/−*^) displayed a metabolic phenotype opposite to that of *Ocn*-null mice, characterized by improved insulin sensitivity, reduced fat mass and increased energy expenditure^125^ (Fig. 4). Taken together, both the gain-of-function and loss-of-function mouse models indicate that OCN plays a key role in the regulation of energy metabolism.^121,125,126^

Further analyses of those genetically modified animal models have revealed the target organs and the mechanisms by which OCN regulates energy expenditure. For adipose, OCN treatment was able to upregulate the expression of *Adiponectin*, an insulin-sensitizing adipokine gene, in white and brown adipose tissues, improve glucose uptake and insulin sensitivity in vivo and suppress the secretion of proinflammatory cytokines in adipocytes in vitro.^38,41,42,121,127^ In steatotic livers of *Ocn*-null mice, impaired insulin sensitivity, increased fat accumulation and inflammation supported the effects of OCN on insulin sensitivity and lipid accumulation.^41,128^

However, OCN supports muscle function during exercise in part through the release of IL-6, the first myokine found to be rapidly released into blood during exercise, enhancing glucose and fatty acid uptake into myofibers.^40,129,130^ Increased IL-6 levels promote the production of bioactive OCN by increasing osteoclast activity through the regulation of RANKL expression in osteoblasts. Hence OCN and IL-6 might have additional functions in mediating the cross talk between bone and skeletal muscle by modulating adaptation to exercise.^40^

Reduced circulating insulin in *Ocn*-deficient mice is explained by a dual-action of OCN on pancreatic β-cells, whereby it increases insulin synthesis and secretion and on the other hand promotes β-cell proliferation.^121,123,131^ Co-culture assays indicate that OCN secreted by osteoblasts promoted β-cell proliferation, insulin secretion and sensitivity.^42^ A separate cell-based assay using isolated pancreatic islets showed that recombinant OCN promoted the expression of the insulin genes, *Ins1* and *Ins2*, and cell-cycle regulators *Ccnd1*, *Ccnd2*, and *Cdk4*, which are known to have a positive action on proliferation^41^ (Fig. 4).

Together, these observations and studies suggest that the effects of OCN on obesity and insulin resistance could be a result in part of its capacity to promote insulin sensitivity in the liver and adipose tissue, energy expenditure in muscle and insulin production in the pancreas, and to upregulate expression of functional genes in the pancreas, muscle and adipose tissue.

### Regulation of male fertility by OCN

As most hormones have multiple functions, an important question is whether OCN has endocrine functions other than regulating energy metabolism. Cell-specific loss-of-function and gain-of-function models demonstrated that osteoblast-derived OCN promoted fertility in male mice. Co-culture assay showed that osteoblasts promoted testosterone synthesis in Leydig cells of the testes, but not estrogen production in the ovaries^43^ (Fig. 4). In addition, the reproductive function of OCN was also found in humans.^132^ These studies provide evidences that interaction between bone and the reproductive system is not limited to regulation of bone remodeling by the gonads but also a positive feedback regulation on reproduction by bone-derived hormones.

### Regulation of cognition by OCN

Following a search for other target organs of OCN, a docile phenotype of *Ocn*-null mice came into notice.^44^ The docility, or passivity, manifested in both male and female *Ocn*-deficient mice even though OCN only regulates male steroid hormones.^43^ A more rigorous analysis revealed that these mice displayed more severe behavioral phenotypes such as a deficit in spatial learning and memory with decreased synthesis of all monoamine neurotransmitters. Intracerebroventricular infusion of OCN into the brains of *Ocn*-null mice corrected the neurotransmitter deficiency and the defect in cognition. It has been found that OCN affects neurotransmitter synthesis by crossing the blood–brain barrier and binding specifically to serotonergic neurons of the raphe nuclei in the brainstem, neurons of the dopaminergic nucleus of ventral tegmental area in the midbrain and neurons of the CA3 region in the hippocampus. Besides a lack of neurotransmitter synthesis, histological analysis showed that the hippocampus of *Ocn*-null mice were hypoplastic. OCN favors hippocampal development by preventing neuronal apoptosis^44,114^ (Fig. 4).

Thus, these results demonstrate that bone has a significant influence on neurotransmitter synthesis, hippocampus development and brain cognitive functions. This study provides a good illustration of the regulation of the brain by skeleton-derived hormones.

### GRPC6A: a putative receptor for OCN

The mediator of GluOCN activity in the targeted tissues was recognized by at least one specific receptor G-protein-coupled receptor class C group 6 member A (GPRC6A) (Fig. 4), generally described as a cation and amino acid sensing receptor.^133,134^ The fact that defects in insulin secretion, glucose tolerance and male reproductive abnormalities in *Gprc6a*^*−/−*^ mice were phenocopies of *Ocn*-null mice indicated that GPRC6A was the receptor for OCN at least in pancreatic β-cells and testis Leydig cells.^135^ Specific inactivation of GPRC6A in the pancreas resulted in reduced β-cell proliferation and decreased insulin secretion, which was similar to that of Ocn-deficient mice.^136,137^ Addition of OCN-induced insulin secretion and β-cell proliferation was abrogated in *Gprc6a*^*−/−*^ islets, additionally suggesting that GPRC6A is the receptor for OCN in β-cells.^136^ Ex vivo evidence showed that OCN bound and activated GPRC6A in β-cells.^137^ These data identified that OCN functions as an endocrine hormone in the pancreas through GPRC6A (Fig. 4).

Stimulation of testosterone secretion by OCN in Leydig cells charts a bell-shaped curve, which is observed when ligands bind to a G-protein-coupled receptor. Furthermore, treatment of the Leydig cells with OCN-induced activation of cAMP, a second messenger of G-protein-coupled receptors. GPRC6A was only expressed in the Leydig cells of the testes and not in follicular cells of the ovary.^43,114^ Furthermore, mutation and polymorphisms of the human *GPRC6A* gene was associated with insulin resistance and failure of testis function, which were also observed in *Ocn*-deficient mice.^132,138^ These facts together confirm that GPRC6A transduces OCN signals in the testes (Fig. 4). Both the *Gprc6a*^*−/−*^ and *Ocn*-null mice show increased fat mass, but the receptor for OCN has not yet been identified in adipocytes.^139^ GPRC6A was not found to be expressed in the brain, and *Gprc6a*^*−/−*^ mice were found to have normal neurotransmitter synthesis, hippocampal development, and cognitive function.^114^ The finding that OCN-GPRC6A signaling in myofibers is required for adaptation to exercise indicates that GPRC6A is the receptor for OCN in muscle^40^ while receptor (s) for OCN in the brain, adipose tissue and liver still require identification (Fig. 4).

### LCN2

Study of the endocrine functions of OCN in the regulation of energy metabolism posed a question as whether additional bone-derived hormones existed which could affect energy metabolism.^139^ Food intake increased in mice that had undergone osteoblast ablation while additional administration of OCN did not further affect the intake, supporting the hypothesis that additional bone-derived hormones may exist and contribute to the regulation of appetite and therefore to the regulation of energy metabolism.^140^ Osteoblast-specific knockdown of *Foxo1*, a transcription factor regulating osteoblast function, displayed improved energy metabolism, only in part due to the activation of OCN.^141^ *Lcn2* was found to be one of the genes that underwent the greatest increase in expression in *Foxo1*-deficient osteoblast,^45^ suggesting that LCN2 could be an additional osteoblast-secreted molecule involved in regulating energy homeostasis downstream of FOXO1.

LCN2, also known as neutrophil gelatinase-associated lipocalin (NGAL), is a small commonly secreted protein with a hydrophobic ligand-binding pocket.^142,143^ Analysis of LCN2 in all tissues showed specific expression of LCN2 in bone. Mice that lacked *Lcn2* in osteoblasts (*Lcn2*^*osb−/−*^), but not in adipocytes (*Lcn2*^*fat−/−*^), showed decreased glucose tolerance, insulin sensitivity, and secretion and conversely, an increase in food intake and body weight in mice lacking *Lcn2* in adipocytes but not osteoblasts (Fig. 4). The number and size of pancreatic islets, in addition to mass and proliferation of β-cells, were also reduced in *Lcn2*^*osb−/−*^ mice. In vitro assays established that LCN2 directlly acted on β-cell proliferation and insulin secretion (Fig. 4).^45^

The increased food-intake phenotype of *Lcn2*^*osb−/−*^ mice and the finding that exogenous LCN2 could suppress food intake in mice suggested it has an anorexigenic function. LCN2 was not expressed in the hypothalamus but shown to regulate food intake by crossing the blood–brain barrier and directly activating cAMP signaling in the hypothalamus following intraperitoneal delivery or intracerebroventricular infusion of LCN2 in *Lcn2* global deleted (*Lcn2*^*−/−*^) mice. Screening of all hypothalamic pathways that affect appetite identified MC4R signaling as the only pathway that was altered in *Lcn2*^*osb−/−*^ mice. LCN2 was subsequently found to bind to the neurons of the paraventricular nucleus of the hypothalamus (PVH) and ventromedial nucleus of the hypothalamus (VMH), where MC4R is expressed and activates MC4R-dependent anorexigenic signaling^45^ (Fig. 4).

Studies of LCN2 identify a novel mode of endocrine regulation of energy metabolism by bone, which occurs through the control of appetite. Consequently, the role of bone in regulating energy homeostasis may provide new insights into the pathogenesis of those disorders: an inversely correlation exists between serum levels of LCN2 and body weight in addition to glycated hemoglobin (HbA1c) in type 2 diabetes patients^45^ and LCN2 recently emerged as a potential clinical biomarker in multiple sclerosis and age-related cognitive decline.^144^ However, the biological function of LCN2 secreted by osteoblasts requires validation in humans with more convincing clinical data.

### Conclusions and perspectives

Local molecular signaling in the control of bone development and remodeling has been extensively studied, while the crosstalk between the bone cells mediated by the secreted proteins has begun to draw consideration. Bone marrow MSCs are multi-potent and can give rise to several distinct cell types including osteoblasts, adipocytes, and chondrocytes. Among these, adipocytes have particular relevance to bone homeostasis.^145^ An imbalance in osteoblast and adipocyte differentiation can result in fatty bone marrow and bone loss due to aging or diabetes mellitus. Bone marrow adipocytes can secrete a spectrum of biologically active molecules such as Leptin and Adiponectin to influence the development and function of osteoblasts and osteoclasts. Further descriptions of the complex influences of leptin and adiponectin on osteoblasts and osteoclasts are described in the following reference.^146–153^ Furthermore, the osteoblasts and osteocytes derived-FGF2 can inhibit the differentiation of adipocytes from bone marrow mesenchymal progenitors,^154^ while the impact of osteoclasts on bone marrow adipocytes is still unclear. As increased marrow adipogenesis associated with decreased bone mass is well known clinically in age-related osteoporosis, further research into the secretion and function of hormones in the bone cells and adipocytes and the contribution of bone marrow adipocytes to the global regulation of energy metabolism will prove to be of great importance.^155^ Overall, a more detailed understanding of the complicated communications between the bone cells, osteoblasts, osteocytes, and osteoclasts, together with the adipocytes, will further the development of treatments for osteoporosis and other bone metabolic diseases.^2,4^

Understanding of the endocrine actions of the skeleton through mouse models and human genetic disorders has revealed distinct mechanisms regulating whole-body energy homeostasis. The next frontier is the discovery of other bone-derived factors that systematically mediate endocrine function.^46^ Large-scale genetic screening combined with bio­informatic prediction and metabolomics might be a powerful approach to efficiently identify such factors. It should be noted that the endocrine factors from bone currently recognized are all secreted by osteoblasts or osteocytes while the role of osteoclasts in the regulation of other organs and whole-body energy balance is still undiscovered. Alternatively, once their role in metabolism is established, it is likely that secretory factors such as OCN and LCN2 could become pharmacological targets in the treatment of obesity, diabetes mellitus or other metabolic diseases. The up-stream signaling involved in the regulation of the secretion and function of those factors should also be taken into account during the target validation and drug discovery stage.
